# Effect of Ultraviolet Radiation on the Enzymolytic and Biomechanical Profiles of Abdominal Aortic Adventitia Tissue

**DOI:** 10.3390/jcm13020633

**Published:** 2024-01-22

**Authors:** Emil-Marian Arbănaşi, Eliza Russu, Eliza-Mihaela Arbănaşi, Constantin Claudiu Ciucanu, Adrian Vasile Mureșan, Shuko Suzuki, Traian V. Chirilă

**Affiliations:** 1 Doctoral School of Medicine and Pharmacy, George Emil Palade University of Medicine, Pharmacy, Sciences and Technology of Targu Mures (UMFST), 540142 Targu Mures, Romania; emilarbanasi1@gmail.com (E.-M.A.); arbanasi.eliza@gmail.com (E.-M.A.); claudiociucanu@gmail.com (C.C.C.); 2 Clinic of Vascular Surgery, Mures County Emergency Hospital, 540136 Targu Mures, Romania; adrian.muresan@umfst.ro; 3 Department of Vascular Surgery, George Emil Palade University of Medicine, Pharmacy, Science, and Technology of Targu Mures, 540139 Targu Mures, Romania; 4 Centre for Advanced Medical and Pharmaceutical Research (CCAMF), George Emil Palade University of Medicine, Pharmacy, Science, and Technology of Targu Mures, 540139 Targu Mures, Romania; shuko.suzuki@qei.org.au (S.S.); traian.chirila@qei.org.au (T.V.C.); 5 Queensland Eye Institute, South Brisbane, QLD 4101, Australia; 6 Faculty of Medicine, George Emil Palade University of Medicine, Pharmacy, Science, and Technology of Targu Mures, 540139 Targu Mures, Romania; 7 School of Chemistry and Physics, Queensland University of Technology, Brisbane, QLD 4001, Australia; 8 Australian Institute of Bioengineering and Nanotechnology (AIBN), University of Queensland, St. Lucia, QLD 4072, Australia

**Keywords:** abdominal aortic aneurysm, tunica adventitia, enzymatic degradation, ultraviolet-A irradiation, mechanical properties

## Abstract

Background: The abdominal aortic aneurysm (AAA) is defined as an increase in aortic diameter by more than 50% and is associated with a high risk of rupture and mortality without treatment. The aim of this study is to analyze the role of aortic adventitial collagen photocrosslinking by UV-A irradiation on the biomechanical profile of the aortic wall. Methods: This experimental study is structured in two parts: the first part includes in vitro uniaxial biomechanical evaluation of porcine adventitial tissue subjected to either short-term elastolysis or long-term collagenolysis in an attempt to duplicate two extreme situations as putative stages of aneurysmal degeneration. In the second part, we included biaxial biomechanical evaluation of in vitro human abdominal aortic adventitia and human AAA adventitia specimens. Biomechanical profiles were examined for porcine and human aortic tissue before and after irradiation with UV-A light (365 nm wavelength). Results: On the porcine aortic sample, the enhancing effect of irradiation was evident both on the tissue subjected to elastolysis, which had a high collagen-to-elastin ratio, and on the tissue subjected to prolonged collagenolysis despite being considerably depleted in collagen. Further, the effect of irradiation was conclusively demonstrated in the human adventitia samples, where significant post-irradiation increases in Cauchy stress (longitudinal axis: *p* = 0.001, circumferential axis: *p* = 0.004) and Young’s modulus (longitudinal axis: *p* = 0.03, circumferential axis: *p* = 0.004) were recorded. Moreover, we have a stronger increase in the strengthening of the AAA adventitia samples following the exposure to UV-A irradiation (*p* = 0.007) and a statistically significant but not very important increase (*p* = 0.021) regarding the stiffness in the circumferential axis. Conclusions: The favorable effect of UV irradiation on the strength and stiffness of degraded aortic adventitia in experimental situations mimicking early and later stages of aneurysmal degeneration is essential for the development and potential success of procedures to prevent aneurysmal ruptures. The experiments on human normal and aneurysmal adventitial tissue confirmed the validity and potential success of a procedure based on exposure to UV-A radiation.

## 1. Introduction

An aneurysm can be described as the regional dilation (as a ‘bulge’ or ‘sac’) of a blood vessel involving its whole wall thickness, which is the result of multifactorial and complex degenerative processes leading to progressive and irreversible loss of the normal structural and mechanical characteristics of the wall. The aneurysms develop commonly in arteries, and the abdominal aortic aneurysm (AAA) is the most frequent in humans. Its sudden and unpredictable rupture at the bulge’s level unleashes extensive bleeding, which is associated with high mortality. There is a vast literature dedicated to the pathophysiology of AAA. Besides inflammatory cell infiltration and depletion of medial smooth-muscle cells, the ongoing remodeling of extracellular matrix (ECM) in the arterial wall is a prominent feature of the pathomechanism, and it is believed that the enzymolysis of medial and adventitial ECM proteins (mostly elastin and collagen) associated with their impaired remodeling plays a critical role in the pathogenesis of AAA [1,2,3,4,5,6,7,8,9]. A range of proteinases are involved in such degradation processes, including serine proteinases (elastases, chymases), matrix metalloproteinases, disintegrin metalloproteinases, and cysteine/serine/aspartyl proteinases (cathepsins, granzymes).

Surgical procedures have been developed for preventing aneurysmal rupture and for carrying out the emergency repair of ruptured vessels [10], based on the exclusion of the bulge from the vascular system. Although associated with improved levels of clinical success, there remains the risk of serious post-operative complications, implying that less invasive procedures would be advantageous. We have suggested that by strengthening mechanically the aneurysmal wall, its rupture can be delayed indefinitely, showing that by exposing the aortic tunica adventitia to ultraviolet A (UV-A) radiation in the presence of photosensitizers, its strength and stiffness were enhanced as a result of collagen crosslinking [11,12,13]. We have also reported [14] a similar effect on the excised whole-thickness saphenous venous wall, which may open new avenues for preventing the neointimal hyperplasia-induced vein graft stenosis. These results are the first to suggest the potential application of UV-induced exogenous collagen crosslinking to vascular tissue for the augmentation of its mechanical characteristics.

Given the involvement of enzymolysis in AAA, the selective proteolytic digestion of the main proteins in the arterial wall matrix (elastin, collagen) has been employed to investigate their fate in aneurysmal degeneration [15,16,17,18,19,20,21,22,23,24,25,26]. Although a number of proteinases may be present and/or involved in these degenerative processes, such studies have mostly used elastase and collagenase. A consensus of these studies [11,12] is that tunica adventitia prevents overstretching and rupture of the wall thanks to its collagen network, which is further supplemented by compensatory remodeled “repair” collagen during aneurysmal degeneration, while tunica media is chiefly responsible for the mechanical properties of the whole healthy aortic wall. Our own studies on collagenolysis [12] and elastolysis [13] of porcine abdominal aortic adventitia have shown that mechanical properties of selectively degraded tissue change in accordance with the intrinsic mechanical characteristics of collagen and elastin. Being about 1000-fold stiffer and 6000-fold stronger than elastin, the mammalian collagen provides strength and rigidity to tissues, while elastin, which is 10 times more extensible than collagen but less strong, provides elevated strain, compliance, and recoil properties [27,28]. Therefore, it is not surprising that collagenolysis of adventitial tissue led to loss of collagen and physical collapse. However, short-term elastolysis resulted in an enhancement of strength and stiffness [13,24], which can be attributed to an increase in the collagen-to-elastin ratio. On the contrary, longer elastolysis led to mechanical weakening of the adventitia caused likely by the degradation of collagen, which can be digested not only by collagenase but also by elastase, as discovered some decades ago [29] and confirmed subsequently [13,30,31]. Our experiments [12,13] have also shown that irradiation with UV-A rays strengthened mechanically the degraded adventitial tissue, assumed to be the result of photochemical crosslinking of residual collagen.

In the present work, we report a unified view of the exposure of the porcine and human abdominal aortic adventitia and the adventitia of the abdominal aortic aneurysmal wall to UV-A irradiation to analyze the efficiency of photocrosslinking of adventitial collagen fibers in strengthening the aneurysmal wall.

## 2. Materials and Methods

### 2.1. Materials

Riboflavin 5′-phosphate monosodium salt (RF) was supplied by Cayman Chemicals (Ann Arbor, MI, USA). Its solution in saline (0.1% *w*/*v*) was used as a photoinitiator for the radiation-induced collagen crosslinking. Elastase from porcine pancreas (Lot No. U1122327529), with an activity of 80 U/mg, double-crystallized, was purchased from MP Biomedicals, LLC (Solon, OH, USA). Collagenase from Clostridium histolyticum (Product No. C2674), with an activity of 389 U/mg, was supplied by MilliporeSigma (St Louis, MI, USA). All other substances and agents were supplied by MilliporeSigma. Water of high purity (Milli-Q or equivalent) was used in the experiments.

#### 2.1.1. Porcine Abdominal Aortic Adventitia Sample

The porcine aortas were obtained from Highchester Meats Pty Ltd. (Gleneagle, Queensland, Australia), an abattoir unit operating under the Australian Code of Practice of Animal Welfare Standards for Livestock Processing Establishments. Aortic segments were harvested on the day of sacrifice from the cadavers of 20-week-old pigs (the variety “beacon pigs”). The animals were sacrificed exclusively for commercial purposes, and the aortas would have been discarded if not acquired for this study.

**Figure 1:**
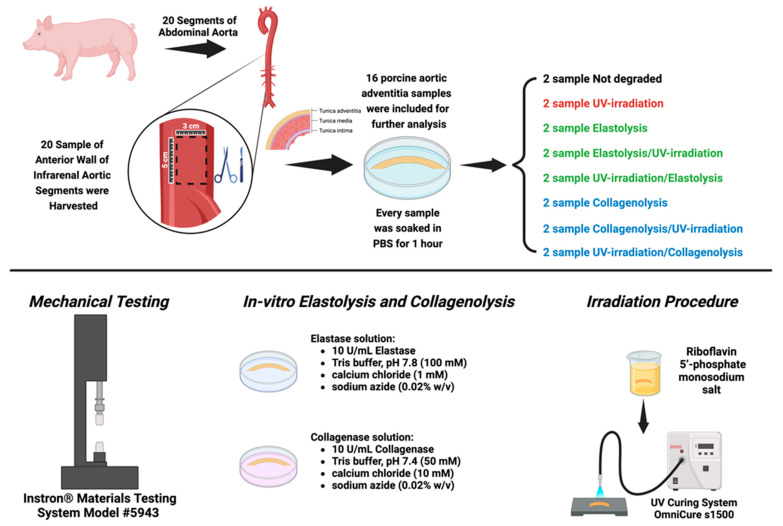
Graphic representation of the design of the experimental study performed on the porcine aortic adventitia. Created with BioRender.com.

Twenty aortic segments selected from the abdominal region were used in this study. They were stored in a freezer (−80 °C) and thawed when needed. The adventitial specimens were prepared as previously reported [11], by removing the extraneous fat and connective tissue from the external surface of the vessel, initiating a dissection at a corner with a surgical scalpel, and carefully peeling off the adventitia by hand. Eight sets of 2 adventitial specimens (5 × 3 cm each) were soaked in phosphate-buffered saline (PBS) for 1 h, and each was placed in a Petri dish (6-cm diameter). Thus, in the end, only 16 porcine aortic adventitia samples were analyzed and included in this study. One set of 2 specimens was employed as an untreated control from the previously mentioned samples, and another set was irradiated only. The remaining samples were divided into 6 sets of 2 specimens each: 3 sets for the collagenolysis protocol and 3 sets for the elastolysis protocol (Figure 1). We aimed at studying adventitial tissue that was significantly depleted of either elastin or collagen. As previously shown [12,13], the first could be obtained by short-term elastolysis, and the second by long-term collagenolysis.

#### 2.1.2. Human Abdominal Aortic Adventitia Sample

**Figure 2:**
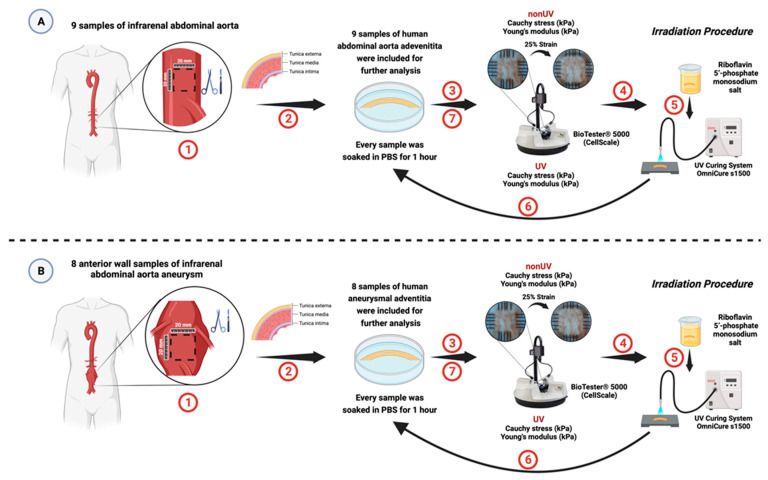
Graphical representation of the experimental study design that was conducted on human aortic adventitia samples. The study stages were similar for samples taken from the normal infrarenal aorta (**A**) at autopsy and those taken intraoperatively from patients with AAA (**B**). The specimens were harvested first (1), from which the adventitia was later prepared and separated (2). After that, the samples were rested in PBS for one hour at room temperature before analyzing the biomechanical profile at 25% strain (3). Further, the samples were then soaked in RF for 30 min (4) and irradiated externally with an intensity of 45 mW/cm^2^ for 10 min (5). Following irradiation, the samples were rested in PBS for one hour (6) and then reanalyzed for their biomechanical profile using the same protocol used previously (7). Created with BioRender.com.

In this study, we enrolled 9 samples of the anterior wall, measuring 20 × 20 mm, collected at the level of the abdominal aorta during the autopsies of 9 subjects aged between 42 and 87 years with sudden cardiac death (confirmed less than 24 h before) performed at the Institute of Forensic Medicine in Targu Mures, Romania. After harvesting, the samples were immediately stored in PBS and taken to the laboratory, where they were stored at 4 °C until further processing. In addition, from each aortic wall sample, we carefully removed the tunica adventitia, which we later processed with the help of a scalpel into 10 × 10 mm samples for determination of a biaxial biomechanical profile and analyzed it in less than 6 h (Figure 2A). Moreover, regarding the subjects, we recorded demographic data (age and sex), anthropometric data (height, weight, and body mass index), and the respective length and diameter of the abdominal aorta. The length was measured from the level of the right renal artery to the level of the aortic bifurcation, and the diameter was quantified as the average value of 3 determinations specific to the aortic segment immediately below the emergence of the renal arteries, the aortic segment before the bifurcation, respectively, at its halfway point, which was measured using a digital caliper.

#### 2.1.3. Human Abdominal Aortic Aneurysmal Adventitia Sample

In this experimental study, we analyzed 8 anterior aneurysmal wall samples taken intraoperatively from patients with AAA, hospitalized in the Vascular Surgery Clinic of the Mures County Emergency Hospital between March and September 2023. The harvested samples were immediately stored in PBS and transported to the laboratory where they were analyzed within 6 h. In the laboratory, 20 × 20 mm specimens were prepared from each wall, and 10 × 10 mm tunica adventitia samples were carefully processed and included in the analysis protocol, as shown in Figure 2B. Additionally, we collected demographic data, including age and sex, BMI, maximum diameter of the AAA, and the presence of common risk factors such as hypertension, diabetes, active smoking, and AAA rupture for each patient.

### 2.2. In-Vitro Elastolysis

An elastase solution containing 10 U/mL was prepared by diluting the original enzyme solution with an aqueous solution made up from Tris buffer with pH 7.8 (100 mM), calcium chloride (1 mM) and sodium azide (0.02% *w*/*v*). Portions (6-mL each) of this elastase solution were added to the dishes to cover the tissue samples in three of the sets. The dishes were then shaken at 160 rpm for 1 h at 37 °C. Three sets of 2 specimens each were processed. One set was subjected to elastolysis only; the second set was subjected to elastolysis and then irradiated; the third set was irradiated and then subjected to elastolysis. Following elastolysis, the samples were rinsed three times in cold water. Prior to mechanical testing, all specimens were kept in PBS at room temperature.

### 2.3. In-Vitro Collagenolysis

Three sets of adventitial specimens were subjected to collagenolysis. A solution containing 10 U/mL collagenase was prepared in Tris buffer (50 mM, pH 7.4), which contained calcium chloride (10 mM) and sodium azide (0.02% *w*/*v*). To each dish, 6 mL of this solution was added to cover the tissue samples. The dishes were shaken at 160 rpm for 20 h at 37 °C. One set was subjected to collagenolytic digestion only, another set was first digested and then irradiated, while the third set was first irradiated and then digested. The residual adventitial samples in each dish were rinsed in cold water with gentle shaking. Prior to mechanical testing, all samples were stored in PBS.

### 2.4. Irradiation Procedure

The rectangular samples of porcine aortic adventitia and square samples of human aortic adventitia to be irradiated were first soaked in the RF solution for 30 min at room temperature. Each specimen was then exposed to UV-A radiation (at a wavelength of 365 nm) generated by a UV Curing System OmniCure 1500 (Excelitas Technologies Corp., Waltham, MA, USA). The localization of irradiance spots on the target was monitored with a radiometer Dymax ACCU-CAL 50 (Dymax Corp., Torrington, CT, USA). The required value for the irradiance was obtained by adjusting the distance between the radiation source and the target. Each side of the specimens was exposed to an irradiance of 45 mW/cm^2^ for 10 min, corresponding to a fluence of 27 J/cm^2^. Furthermore, for the human adventitia specimen, we exposed only the external side, taking into account the clinical applicability, with the same parameters mentioned previously.

### 2.5. Mechanical Testing

#### 2.5.1. Protocol for Porcine Adventitia Samples

An Instron^®^ Materials Testing System Model #5943 (Instron, Norwood, MA, USA), equipped with a 50-N load cell, was employed to obtain the stress–strain plots in the uniaxial mode. Two strips (1 × 3 cm) were cut from each adventitial specimen, and the plots were recorded for a total of four strips. Representative stress–strain plots presented here were selected based on previously measured [11,12,13,14] values for Young’s modulus and ultimate stress.

#### 2.5.2. Protocol for Human Adventitia Samples

For the biaxial biomechanical analysis, we used the BioTester^®^ 5000 (CellScale, Waterloo, ON, Canada), from the Laboratory of Regenerative Medicine of the CCAMF within the UMFST “George Emil Palade”, Targu Mures, Romania. The biotester is equipped with four 23-N load cells, for which we used four rakes armed with 8.5 mm active parts. Based on the size of the rakes, an initial distance of 8.5 × 8.5 mm was preset and tracked the force–displacement graph for a 25% stretch of each sample at a speed of 1%/s for a total of 10 cycles, standardized by a 25 s stretch period, followed by 25 s for recovery. Using the data generated by the biotester’s LabJoy 2.0 software (CellScale, Waterloo, ON, Canada), we calculated the Cauchy stress and Young’s modulus using the values from the last cycle, similarly to the formulas presented [11,12,13,14].

### 2.6. Statistical Analysis

For statistical analysis, we used IBM SPSS 28.0.1.0 for macOS (IBM software, Armonk, NY, USA). The data were presented as mean, minimum, maximum, and 95% confidence interval. The difference between the biomechanical parameters before and after exposure to UV-A radiation was calculated with the Mann–Whitney U-test and recognized when the *p*-value was less than 0.05, corresponding to a 95% confidence level. We used the Spearman correlation to analyze the association between the benefit of UV-A radiation treatment and the age of the subjects from which we harvested the human aortic adventitia samples.

## 3. Results

### 3.1. Experiment on Porcine Aortic Adventitia

**Figure 3:**
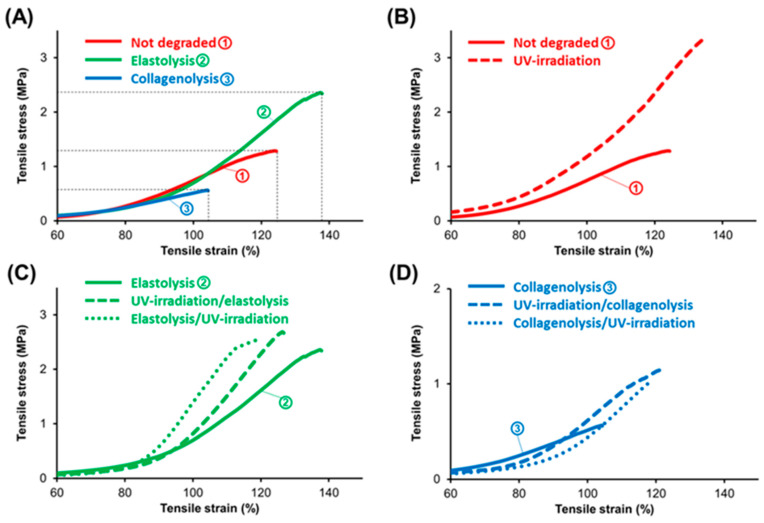
Stress–strain plots of aortic adventitial tissue under uniaxial loading. (**A**). Plots of enzymatically degraded (elastase, collagenase) and non-degraded tissue specimens. (**B**). Plots of non-degraded tissue before and after irradiation. (**C**). Plots of tissue exposed to elastase (10 U/mL, 1 h), irradiated before and after digestion. (**D**). Plots of tissue exposed to collagenase (10 U/mL, 20 h), irradiated before and after digestion. Irradiation was performed with UV-A rays (wavelength 365 nm), at an irradiance of 45 mW/cm^2^, for a duration of 10 min (resulting in a fluence of 27 J/cm^2^).

The stress–strain plots for all the adventitial tissue samples are shown in Figure 3. The master stress–stain profiles in Figure 3A show what was to be expected. Following short-term elastolysis, the tissue became stronger, with an ultimate tensile stress (UTS) around 2.3 MPa (plot “2”), as compared to UTS for the non-degraded tissue of ~1.2 MPa (plot “1”) and ~0.6 MPa for the tissue subjected to collagenolysis (plot “3”). Mechanical augmentation associated with short-term elastolysis is the consequence of a significant drop in the elastin-to-collagen ratio due to fast digestion of the adventitial elastin.

### 3.2. Experiment on the Human Aortic Adventitia

The subjects enrolled in this study had an average age of 71 years, an average weight of 67.7 kg, and a height of 160.3 cm. We also recorded an average value of the aortic diameter of 1.85 cm, between 1.54 cm and 2.26 cm, and, respectively, an average length of 10.38 cm between 8.1 cm and 12.7 cm (Table 1).

**Figure 4:**
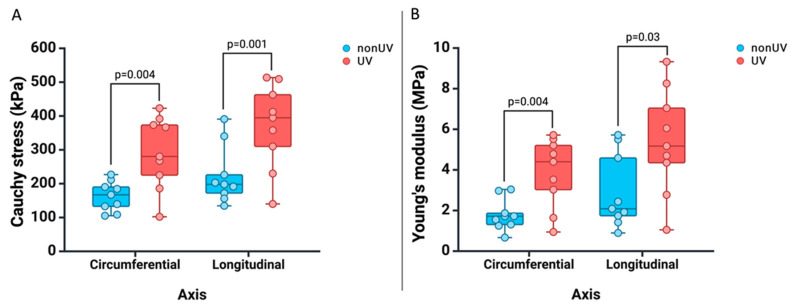
Plots of Cauchy stress (**A**) and Young’s modulus (**B**) at 1.25 stretch ratio in the longitudinal and circumferential directions for the 9 human abdominal aortic adventitia samples suggesting an increase in strength and stiffness post-UVA irradiation for both axes.

Each aortic adventitia sample was analyzed biomechanically biaxially, followed by its treatment with UV-A, and after an hour of rest at room temperature, it was re-analyzed biomechanically. As can be seen in Table 2 and Figure 4, we recorded a post-treatment increase in the strength of the samples, both in the longitudinal axis (Cauchy stress: 370.1 kPa vs. 223.6 kPa, *p* = 0.001) and in the circumferential axis (Cauchy stress: 290.6 kPa vs. 162.9 kPa, *p* = 0.004), respectively, an increase in the stiffness of the samples in the longitudinal axis (Young’s modulus: 5412.8 kPa vs. 2992.2 kPa, *p* = 0.03), as well as in the circumferential axis (Young’s modulus: 3861.2 kPa vs. 1787.3 kPa, *p* = 0.004).

**Figure 5:**
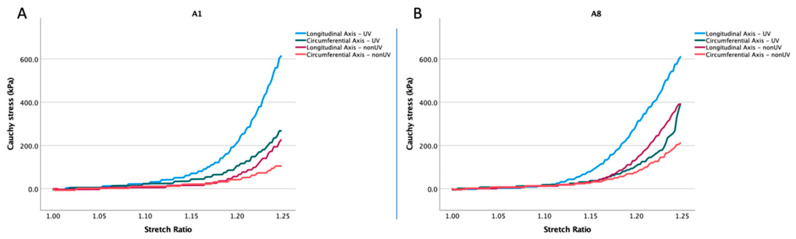
Cauchy stress versus stretch behavior of human abdominal aortic adventitia samples obtained from biaxial mechanical tests indicating a significant increase in mechanical strength post-UVA irradiation: (**A**): sample A1 and (**B**): sample A8.

For a better graphic display, in Figure 5, we presented the stress–stretch plot for two samples, where we recorded an important increase in the biomechanical parameters after UV-A irradiation. Thus, in Figure 5A, for A1, we recorded an increase in Cauchy stress from 226.2 kPa to 613.6 kPa in the longitudinal axis and from 105.2 kPa to 267.38 kPa in the circumferential axis. Also, for A8 (Figure 5B), we recorded an increase in Cauchy stress from 390.88 kPa to 609.65 kPa in the longitudinal axis and from 211.3 kPa to 391.66 kPa in the circumferential axis.

**Figure 6:**
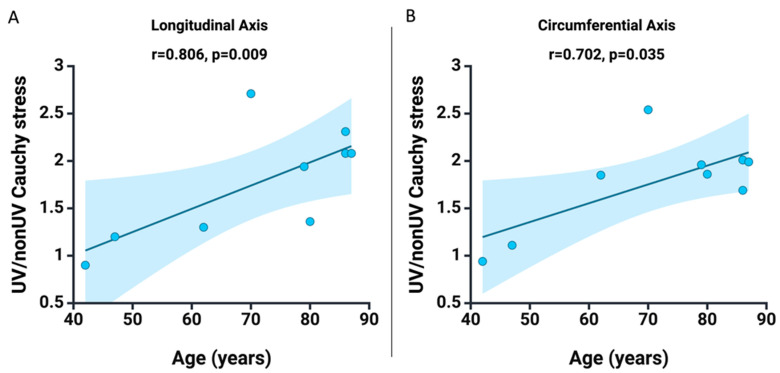
**Spearman** correlation between age and the benefit of exposure to UV-A radiation specific to Cauchy stress for (**A**) the longitudinal axis and (**B**) the circumferential axis. Additionally, the graphs display the fitted line, which is a linear function that describes the data set, and the shaded area indicates the 95% confidence interval.

Further, we observed a tendency to increase the benefit of the treatment proposed in the samples taken from the elderly patients compared to the young ones. Thus, we analyzed the correlation between the benefit of the treatment, represented by the ratio between the Cauchy stress values recorded after UV-A irradiation and the initial ones, and the age of the subjects. As seen in Figure 6, we have a statistically significant positive correlation between the benefit of irradiation and the age of the subjects for the longitudinal axis (r = 0.806, *p* = 0.009) and the circumferential axis (r = 0.702, *p* = 0.035).

### 3.3. Experiment on the Human Abdominal Aortic Aneurysmal Adventitia Sample

AAA anterior wall samples were obtained from six men and two women with a mean age of 75.25 years and a mean maximum aneurysm diameter of 82.62 mm (range 65 to 115 mm). Moreover, six patients were hypertensive, four were diabetic, and five were active smokers. In addition, four patients presented with AAA rupture (Table 3).

**Figure 7:**
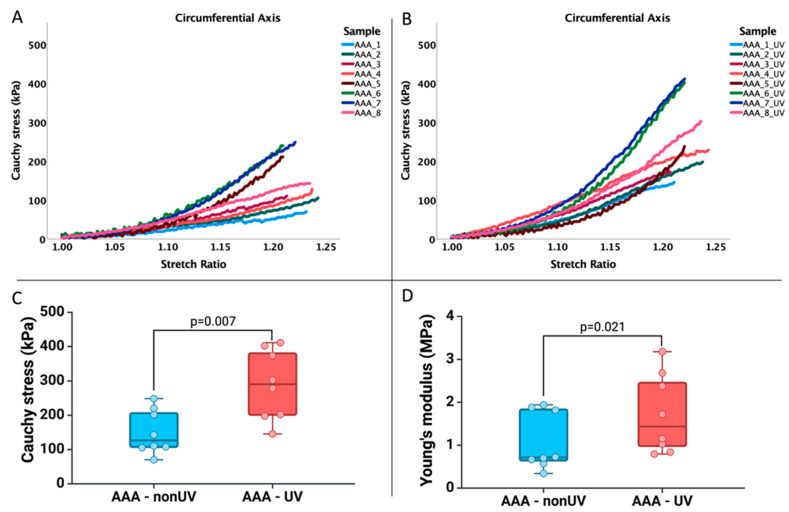
Graphic representation of circumferential axis of (**A**): Cauchy stress versus stretch behavior of all human abdominal aortic aneurysmal adventitia samples, non-UV, (**B**): Cauchy stress versus stretch behavior of all human abdominal aortic aneurysmal adventitia samples following exposure to UV-A irradiation, and (**C**,**D**): the biomechanical profile of samples before and after treatment.

Further, we analyzed the biomechanical profile of the AAA adventitia specimens in the circumferential axis, given that this is the weakest area of the AAA. As seen in Figure 7, we have a stronger increase in the strengthening of the AAA samples following the exposure to UV-A irradiation (*p* = 0.007) and a statistically significant increase (*p* = 0.021), but not very important regarding the stiffness of the AAA samples.

## 4. Discussion

In the current work, we explored the importance of adventitia collagen fibers in the mechanical strength of the aortic wall and the efficacy of their photocrosslinking by UV-A irradiation as a potential treatment of the aneurysm wall. In the first part of the study, we presented the behavior of the enzymolytic profile of porcine aortic adventitia subjected to uniaxial stress and the effect of its irradiation on both normal and enzymatically degraded specimens. Further, we demonstrate the increase in stiffness and strength of the human aortic adventitia following its exposure to UV-A radiation on a group of nine samples taken from the anterior wall of the infrarenal abdominal aorta from nine subjects with sudden cardiac death. However, the main results of this study are validating the results on porcine aorta and human aorta specimens on AAA adventitia samples. For the first time, we demonstrated that following the exposure of the wall to UV-A irradiation, we have a stronger increase in the strengthening of the samples and a statistically significant but not very important increase regarding the stiffness of the samples.

Over the past 10 years, a significant amount of attention regarding AAA has been redirected towards the analysis of the aneurysmal wall from a biomechanical point of view [32,33,34,35,36,37,38,39,40,41] and the attempt to determine a pattern in its behavior in order to subsequently identify molecular mechanisms with modifiable potential, based on which new therapeutic strategies can be developed.

Obtaining results similar to those of this study, Pukaluk et al. [26,32] recently published two articles in which they analyzed the microstructural changes of the human aortic medial layer and adventitia under biaxial loading and recorded an average stretch ratio at failure of 1.31 (between 1.2 and 1.4), a Cauchy stress between 200–600 kPa for the adventitia, and at an average stretch ratio at failure of 1.35 (between 1.26 and 1.4), a maximum Cauchy stress below 400 kPa for the medial layer. These results highlight the adventitia’s biomechanical role as the aortic wall’s last structure of mechanical strength relevance. In contrast, Niestrawska et al. [33] recorded at 1.15 stretch a greater strength of the medial layer compared to the adventitia and the integral aortic wall for the longitudinal and circumferential axes. The conflicting results can be attributed to the age heterogeneity of the subject group from which the samples were taken. Thus, in one of the mentioned studies [32], the average age was 50 years, while in another study [33], it was ~62 years, which suggests that the adventitial contribution to the wall’s biomechanics diminished with age. Based on these observations and on our own results, the need to strengthen mechanically the adventitia becomes evident. This would be possible by applying new therapeutic strategies like that proposed by us. Moreover, a study [34] on tissue collected intraoperatively from AAA patients has shown that the calcified aneurysmal walls displayed a much lower strength and an increased risk of rupture when compared to the fibrous aneurysmal walls group. Considering that most of the aneurysms are calcified, this finding further supports the purpose of our present study to strengthen mechanically the adventitia.

A brief recapitulation of the succession of events occurring in the aneurysmal degeneration [1,2,14,22,28,29,30] may help with interpreting the resulting profiles shown in Figure 3C,D. In the earlier stages of aneurysmal degeneration, the loss of medial elastin and collagen triggers the recruitment of collagen from the outer media and adventitia to cope mechanically with the pulsatile load. As this process becomes less efficient in time, an enhanced turnover follows that is aimed at generating supplementary remodeled “repair” collagen, leading to reinforcement of the adventitia. While the degeneration of medial structures advances unabatedly, it is the adventitia that remains to stand alone against the mechanical weakening of the wall. In time, the ongoing degradation processes suppress its collagen remodeling capacity, and consequently the altered neocollagen is rendered insufficient or/and unsuitable for an efficient contribution to the strength of the wall, and rupture becomes unavoidable. The profiles of the samples subjected to elastolysis and irradiated before or after digestion are shown in Figure 3C. In both cases, there was an enhancing effect on strength (UTS increased from ~2.3 MPa to ~2.5 MPa when irradiated prior to degradation, and to ~2.7 MPa when irradiated after degradation, representing mechanical gains of 20% and 40%, respectively). The stiffness (as Young’s modulus, YM) showed a similar trend: ~5.6 MPa (non-irradiated) compared with ~7.9 MPa (irradiated before degradation) and ~8.4 MPa (irradiated after degradation). We can assume that this experimental situation mimics an early stage in the aneurysmal degeneration when elastin was all but removed from the adventitia. A similar progress was seen in the profiles shown in Figure 3D, which may mimic a later stage when adventitial collagen was significantly depleted. UTS increased from ~0.6 MPa to ~1 MPa when irradiation was carried out after degradation and to ~1.2 MPa when it was carried out prior to degradation, representing 40% and 60% mechanical gains, respectively. An inversion should be noted in Figure 3D when compared to Figure 3C, as the tissue irradiated before degradation became stronger. YM increased from ~1.4 MPa to values around 3.2 MPa for the irradiated samples. The effect of irradiation with UV-A rays on enzymatically degraded adventitial tissue is of relevance to the validity of our proposed method aimed at delaying the rupture of an aneurysmal wall. Figure 3B shows the effect on non-degraded healthy adventitia, as a comparison. The enhancement of mechanical properties is evident.

The above findings are of particular importance, as they show that UV irradiation may be beneficial to a degenerated wall by enhancing its mechanical properties, even when the aneurysmal degeneration is advanced and residual collagen seriously depleted. Mechanical strengthening and stiffening of the adventitia can eventually impede the aneurysmal wall’s rupture. Even though the results of our translational research support the efficiency and feasibility of a new possible therapeutic strategy based on irradiation with UV light, this study has some limitations that shall be discussed. The most important limitation is the absence of histological and immunohistochemical analysis to reveal the impact of irradiation on the extracellular matrix and the structure of the aortic wall, an aspect that will be addressed by us in the next stage when the adventitia will be irradiated in vivo within the frame of a murine AAA model described in the literature [42,43]. Secondly, in order to analyze the relationship between age and therapeutic response on the biomechanical profile of the aortic wall, we propose to harvest a large number of samples from well-defined age ranges. Finally, our results on abdominal aortic adventitia were taken from subjects with an intact aorta, without severe atherosclerotic deposits, and without aneurysmal dilatation. Due to the significant structural differences between the normal and the aneurysmal aortic wall, it is necessary to validate these results on a large sample of the human aneurysmal aortic wall.

## 5. Conclusions

The in vitro mechanical properties of the isolated porcine aortic adventitia are affected significantly by degradation with either elastase or collagenase. Short-term elastolysis, resulting in substantial loss of elastin, leads to the mechanical strengthening of the tissue, which is further strengthened following UV irradiation. Long-term collagenolysis results in considerable weakening of the tissue, which nevertheless responds to irradiation by enhancing mechanical properties. Moreover, we demonstrate the increase in biomechanical parameters of human aortic adventitia specimens following exposure to UV-A irradiation. However, the main results of this study are validating the results on the porcine aorta and human aorta specimens on human AAA adventitia samples, where we demonstrated that following the exposure of the wall to UV-A irradiation, we have a stronger increase in the strengthening of the samples and a statistically significant but not very important increase regarding the stiffness of the samples. The beneficial mechanical effect of UV irradiation on degraded porcine aortic adventitia and on human aortic adventitia samples are relevant for the development of procedures to prevent aneurysmal rupture.

## Figures and Tables

**Table 1:** The demographic, anthropometric characteristics, and the dimensions of the abdominal aorta for the subjects enrolled in the study.

Aorta Sample	Sex	Age	Weight (kg)	Height (cm)	BMI	Aorta Diameter (cm)	Aorta Length (cm)
A1	F	86	55	155	22.8	1.96	10.2
A2	F	70	40	139	20.7	1.7	8.1
A3	M	47	82	176	26.4	1.78	9.5
A4	M	80	90	170	31.1	2.26	12.6
A5	F	79	64	158	25.6	1.84	10.5
A6	M	86	64	161	24.6	1.88	12.7
A7	F	87	63	147	29.1	1.76	9.5
A8	M	62	61	163	22.9	1.54	10
A9	M	42	91	174	30.2	1.94	10.4
Mean value		71	67.7	160.3	25.9	1.85	10.38

**Table 2:** The biomechanical profile of all human abdominal aortic adventitia samples presented, depending on the presence of treatment (UVA irradiation).

Axis	Biomechanical Properties	Treatment	Mean	Minimum	Maximum	95% CI		*p*-Value
						Lower	Upper	
Longitudinal (Ox)	Cauchy stress (kPa)	Non-UV	223.6	134.5	390.8	157.7	289.5	0.001
		UV	370.1	140.1	513.6	273.1	467.01	
	Young’s modulus (kPa)	Non-UV	2992.2	893.5	5718.4	1511.1	4333.3	0.03
		UV	5412.8	1047.6	9325	3412.8	7412.7	
Circumferential (Oy)	Cauchy stress (kPa)	Non-UV	162.9	102.2	226.9	129.2	196.6	0.004
		UV	290.6	105.2	422.9	201.8	372.9	
	Young’s modulus (kPa)	Non-UV	1787.3	666.1	3038	1190.1	2383.6	0.004
		UV	3861.2	941.1	5715.8	2547	5175.4	

**Table 3:** Patient characteristics of the 8 analyzed AAA specimens: demographic data (age and sex), BMI, maximum AAA diameter, hypertension, diabetes, active smoking, and AAA rupture.

Sample	Sex	Age (years)	AAA Diameter (mm)	BMI	Hypertension Yes/No	Diabetes Yes/No	Active Smoking Yes/No	Ruptured Yes/No
AAA_1	M	63	66	22.95	No	No	Yes	No
AAA_2	M	74	75	25.39	Yes	Yes	No	No
AAA_3	M	78	89	20.5	No	Yes	No	Yes
AAA_4	F	72	77	28.4	Yes	Yes	Yes	No
AAA_5	M	80	91	31.4	Yes	No	Yes	Yes
AAA_6	F	79	83	32.05	Yes	No	Yes	Yes
AAA_7	M	81	115	27.99	Yes	No	Yes	Yes
AAA_8	M	75	65	27.05	Yes	Yes	No	No

## Data Availability

Information can be provided upon reasonable request.
